# Interview Findings with International Healthcare Providers on Cultural and Environmental Factors in Personal Protective Equipment Use

**DOI:** 10.1017/ash.2025.367

**Published:** 2025-09-24

**Authors:** JaHyun Kang, Jiyun Ha, Yuna Jang, Patti Grota

**Affiliations:** 1 Seoul National University College of Nursing; 2 Seoul National University; 3 College of Nursing, Seoul National University; 4 Ut Health Of San Antonio

## Abstract

**Background:** The influence of variations in healthcare environments and cultural factors on the appropriate use and effectiveness of personal protective equipment (PPE) remains insufficiently explored. This study aimed to investigate complex PPE-related challenges and identify potential solutions through in-depth interviews with international healthcare providers. **Methods:** Study participants were recruited through invitation emails sent to international conference attendees who showed interest to the researcher, contacts from the researcher’s international networks, and authors of publications on PPE. After obtaining study consent and permission for recording, online interviews were scheduled for one hour per participant, except for one in-person interview. Participants were asked to complete an online pre-survey and, if possible, provide PPE pictures. An $80 incentive was offered unless declined by the participant. Narrative responses were transcribed, reviewed, and analyzed. **Results:** From October 17, 2024, to January 9, 2025, interviews were conducted with 13 participants representing 12 countries (Bangladesh, Brazil, Ethiopia, Ghana, Japan, Malaysia, Mongolia, Saudi Arabia, Singapore, South Korea, United States, and Vietnam). The participants were predominantly doctors (69.2%) and male (61.5%), with an average age of 47 years and an average of 21 years of clinical experience. In beard-growing countries (Bangladesh, Ghana, Malaysia, and Saudi Arabia), men with long beards faced challenges in properly covering them with masks and had to use beard covers. Tropical countries (Bangladesh, Brazil, Ghana, and Ethiopia) often lack air conditioning in most healthcare settings, and healthcare personnel (HCP) frequently experience heat-related issues when wearing PPE. Singapore and Japan showed good PPE compliance due to their collective cultures. In Singapore, it stemmed from shared agreement and rule-following, while in Japan, it was driven by group conformity and avoiding inconvenience to others. In Vietnam, PPE compliance was high as bosses evaluated HCP’ compliance, which could influence bonuses. Political issues impacted COVID-19 responses: in Brazil, parties debated masks; in the U.S., masks were seen as personal freedom; in South Korea, unions resisted lowering PPE standards later in the pandemic. In Saudi Arabia, HCP must complete training with PPE tests, including N95 fit testing, to receive a two-year national certification. **Conclusion:** This study identified PPE use characteristics and challenges across countries, influenced by factors such as emerging infectious diseases response capacity, PPE availability, religion, ethnicity, climate, and social context. Future research needs to include a larger-scale study involving more countries, with adequate samples across healthcare systems and hospitals of various sizes, to further investigate issues related to PPE use.

